# Topical Administration of Manuka Oil Prevents UV-B Irradiation-Induced Cutaneous Photoaging in Mice

**DOI:** 10.1155/2013/930857

**Published:** 2013-05-26

**Authors:** Oh Sook Kwon, Seung Hee Jung, Beom Seok Yang

**Affiliations:** ^1^Department of Integrative Medicine, Korea University Medical School, 126-1 Anam-Dong, Sungbuk-Gu, Seoul 136-705, Republic of Korea; ^2^Chemical Kinomics Research Center, Korea Institute of Science and Technology, 39-1 Hawolgok-Dong, Sungbuk-Gu, Seoul 136-791, Republic of Korea

## Abstract

Manuka tree is indigenous to New Zealand, and its essential oil has been used as a traditional medicine to treat wounds, fever, and pain. Although there is a growing interest in the use of manuka oil for antiaging skin care products, little is known about its bioactivity. Solar ultraviolet (UV) radiation is the primary environmental factor causing skin damage and consequently premature aging. Therefore, we evaluated manuka oil for its effects against photoaging in UV-B-irradiated hairless mice. Topical application of manuka oil suppressed the UV-B-induced increase in skin thickness and wrinkle grading in a dose-dependent manner. Application of 10% manuka oil reduced the average length, depth, and % area of wrinkles significantly, and this was correlated with inhibition of loss of collagen fiber content and epidermal hyperplasia. Furthermore, we observed that manuka oil could suppress UV-B-induced skin inflammation by inhibiting the production of inflammatory cytokines. Taken together, this study provides evidence that manuka oil indeed possesses antiphotoaging activity, and this is associated with its inhibitory activity against skin inflammation induced by UV irradiation.

## 1. Introduction

Manuka tree (*Leptospermum scoparium*) is a small shrub that grows in most parts of New Zealand, and its essential oil has been used for many centuries by the native tribes and the immigrants of New Zealand and Australia to treat wounds, infection, inflammation, fever, and pain. *In vitro* and *in vivo* studies have suggested the oil to contain various bioactive constituents. Among them, beta-triketons are best known to possess high antibacterial and antifungal activities [1–4]. In addition, manuka oil possesses sesquiterpene hydrocarbons [1–3], which show anti-inflammatory and analgesic actions [5]. Manuka oil was also known to contain substantial amount of antioxidant compounds that can protect cell components from the harmful action of free radicals [1, 3, 6]. Currently, its commercial application for a skin care and an antiaging product is increasing. However, scientifically controlled studies about its efficacy against skin aging are still rare.

Solar ultraviolet (UV) radiation is one of the most harmful environmental factors that cause skin damage. Repeated exposure to UV radiation ultimately causes premature skin aging also called photoaging, which is characterized by formation of fine and coarse wrinkles, increased skin thickness, dryness, laxity, and pigmentation [7, 8]. The cellular and molecular mechanisms of UV irradiation-induced photoaging have been studied extensively in the last 2 decades. UV irradiation induces the production of reactive oxygen species (ROS) in skin cells, which is primarily responsible for photoaging [9, 10]. The generated ROS activates cellular signaling pathways to activate kinases such as p38, Jun N-terminal kinase (JNK), and mitogen-activated protein kinase (MAPK) [11, 12]. These kinases ultimately stimulate the transcriptional activities of activator protein-1 (AP-1) and nuclear factor- (NF-) *κ*B [13, 14]. Among the main target genes of these 2 transcriptional factors are the genes encoding for matrix metalloproteases (MMPs) such as MMP-1, MMP-3, and MMP-9 [15]. MMPs degrade collagen fibers in the skin, and collagen fibers are an important part of the connective tissue involved in the maintenance of dermal strength and elasticity. Reduction in collagen fibers and their degradation into fragments are important steps that lead to skin aging. The activation of AP-1 has been suggested to antagonize transforming growth factor- (TGF-) *β* signaling pathway which stimulates the expressions of procollagen genes [16]. UV irradiation also induces skin inflammation, which in turn leads to the release of inflammatory cytokines such as tumor necrosis factor- (TNF-) *α* and interleukin- (IL-) 1*β* [14, 17]. These cytokines can accelerate photoaging by inducing MMP expression in addition to increasing epidermal hyperplasia [18, 19]. Since the ROS generated are the main cause of photoaging due to UV irradiation, various antioxidant compounds have been tested to protect the skin from photoaging. Antioxidants remove free radicals and help repair cellular damage to an extent. Topical applications of various antioxidants such as vitamins C and E, selenium, soy isoflavones, and polyphenolic compounds attenuated photoaging [20–22].

In this study, we evaluated antiphotoaging activity of manuka oil by using UV-irradiated hairless mice since the oil contains antioxidant and anti-inflammatory bioactive chemicals, which we thought would prevent photoaging.

## 2. Material and Methods

### 2.1. Animals and UV-B Radiation

Six-week-old female albino hairless mice (SKH-1) were obtained from the Charles River Laboratory (Wilmington, MA, USA) a week before the experiment. Animals were kept at a temperature of 23 ± 1°C and 50% ± 10% of relative humidity in a specific pathogen-free environment. The mice were divided into 5 groups with 6 mice in each group. The animal experiment was approved by the institutional ethics committee for animal care of Korea Institute of Science and Technology and was conducted in accordance with the guidelines for the care and use of laboratory animals (Institute of Laboratory Animal Resources, 1996) as adopted and promulgated by the National Institutes of Health. For UV-B irradiation, TL20W/01RS UV lamps (Philips, Somerset, NJ, USA) with an emission spectrum between 275 and 380 nm (peak: 310–315 nm) and a Kodak cell filter to remove wavelengths of less than 290 nm (UVC) were used. UV-B irradiation intensity on the mouse skin surface was measured using a UV meter (Waldmann GmbH & Co., Villingen-Schwenningen, Germany). The irradiation intensity at 30 cm from the light source was about 0.5 mW/cm^2^. Initially, the minimal erythema dose (MED) to induce erythema with sharp margins on the dorsal skin of the mice after 48 was defined as 1 MED, which was calculated to be approximately 100 mJ/cm^2^. The mice were exposed to UV light 3 times per week (Monday, Wednesday, and Friday) for a total of 8 weeks. The radiation dose was increased weekly by 1 MED from 1 MED up to 3 MED and then maintained at 3 MED until the end of the experiment. Manuka oil purchased from Coast Biologicals, New Zealand, was diluted at 3 different concentrations of 1%, 5%, and 10% using ethanol, and 150 *μ*L of the solution was topically applied to the dorsal area every day except Sunday for 8 weeks. On the day of UV irradiation, mice were treated after UV exposure.

### 2.2. Evaluation of Skin Thickness and Wrinkle Formation

The dorsal skin of the hairless mice was lifted up by pinching gently, and skin thickness was measured using a caliper (Tokyo, Japan) at weeks 0, 2, 6, and 8 of the study period. Wrinkle formation was evaluated visually by 3 trained graders according to the scaling grade described by Bissett et al. at weeks 0, 2, 6, and 8 during the study period [23]. The graders were blinded to the radiation dose administered to the mice. Before the animals were killed at the end of the 8-week study period, skin surface impressions for skin wrinkle replicas were prepared by applying silicon rubber (Silflo Dental Impression Material, Flexico Developments, Stevenage, Hertfordshire, UK) to the dorsal skin of the unstrained mice. Wrinkle shadows from the impression replicas were produced by illuminating the replica on a horizontal stand with a light source angled at 35°, and the images were recorded and analyzed using Skin-Visiometer VL 650 and its software (Courage & Khazaka, Cologne, Germany). The average length, average depth, and the % area of wrinkles were determined as described previously [24].

### 2.3. Histological Analysis and Collagen Staining

The mice were killed by cervical dislocation under anesthesia at the end of the experiment. For histological analyses, skin specimens were obtained from the central dorsum and fixed in 4% formalin before being embedded in paraffin. Five-micrometer thick slices were sectioned from the paraffin-embedded specimens and were deparaffinized and processed by haematoxylin and eosin staining for histological analysis and Masson trichrome staining for the visualization of collagen fibers. Five randomly chosen fields per stained section were photographed under a light microscope with 200x magnification. By using the photographs, epidermal thickness was measured as the distance from the basement membrane in the interfollicular epidermis to the bottom of the stratum corneum. Furthermore, the integrated optical density of the collagen fibers was measured using Image Pro Plus 6.0 software.24, and the % relative collagen density was calculated by normalizing to 100% density value of normal skin treated with a vehicle.

### 2.4. Reverse Transcriptase Polymerase Chain Reaction (RT-PCR)

Total RNA was isolated from skin biopsy samples stored in liquid nitrogen using Triazol (Invitrogen, Carlsbad, CA, USA) according to the manufacturer's protocol and its concentration was determined by measuring the absorbance at 260 and 280 nm. The quality and quantity of the extracted RNA were confirmed by electrophoresis in 1% denaturing agarose gel. The oligonucleotide primer sequences for RT-PCR analysis to estimate the murine m-RNA level of MMP-1, MMP-3, and GAPDH were used as described previously (MMP-1; 5′-ccaggtgtggggtgcctgat-3′ and 5′-caaacctgggcctggctgga-3′, MMP-3; 5′-tagcaggttatcctaaaagca-3′ and 5′-ccagctattgctcttcaat-3′, and GAPDH; 5′-cccactaacatcaaatgggg-3′ and 5′-acacattgggggtaggaaca-3′) [25, 26]. For RT-PCR reactions, 2 *μ*g of total RNA was incubated with 50 ng of random hexamers, and 200 U of reverse transcriptase (Invitrogen, Carlsbad, CA, USA) for 50 min at 50°C, and the reaction was terminated at 85°C for 5 minutes. A 1 : 10 dilution of the reaction mixture containing c-DNA was subjected to PCR amplification using 20 pmol of primers and 1 U of amfiSure Taq DNA polymerase (Gendepot, Barker, TX, USA) with the cycling program: 1st cycle, 15 min at 94°C, next 32 cycles; 30 s at 94°C, 30 s at 56°C, and 30 s at 72°C and the final cycle, 7 min at 72°C. 20 *μ*L of the PCR product was separated by electrophoresis on 1.5% agarose gel and stained with ethidium bromide. 

### 2.5. Preparation of the Total Protein Extract and Enzyme-Linked Immunosorbent Assay for Cytokines

Biopsied tissues obtained from the mice that were killed were stored in liquid nitrogen. The frozen tissues were ground into powder and homogenized in 1 mL of lysis buffer containing 20 mM of Tris (pH: 7.5), 150 mM of NaCl, 1 mM of EDTA, 1% Triton-X-100, and protease inhibitor cocktail (Roche, Indianapolis, MN, USA). The samples were then sonicated 5 times for 10 s, and the undissolved pellet was removed by centrifugation at 10,000 g for 10 min at 4°C. The supernatant was assayed for TNF-*α* and IL-1*β* by using an enzyme-linked immunosorbent assay (ELISA) kit purchased from eBioscience (San Diego, CA, USA). Each measurement was done in triplicate according to the manufacturer's instructions.

### 2.6. Immunohistochemical Staining of Macrophages

5 *μ*m-slices of skin tissue section were deparaffinized and rehydrated, then treated with 3% hydrogen peroxide solution for 5 min, and blocked by using 1% normal sera. The slides were incubated with rabbit monoclonal antibody of CD163 (Abcam, Cambridge, UK) after 1 : 100 dilution, followed by incubation with goat antirabbit antibody conjugated with HRP (1 : 200 dilution; Gendepot, Baker, TX, USA). The staining signals on the tissue slides were detected with DAB+ Substrate—Chromogen (DAKO, Carpinteria, CA, USA) following the manufacturer's instructions. Thereafter, counterstaining was carried out with hematoxylin. Five randomly chosen fields per each stained section were photographed under a light microscope with 200x magnification and the stained macrophages were counted, followed by calculating the average number.

### 2.7. Data Analysis

Data were presented as mean ± SEM for each treatment group and plotted using SigmaPlot software (Systat Software Inc., San Jose, CA, USA). The SPSS for Windows version 12.0 was used for the statistical analysis. Unpaired Student's *t*-test was used to compare differences between 2 groups. Statistical significance between the different doses of manuka oil to inhibit the increase of skin thickness and wrinkle grading was analyzed by using repeated-measures analysis of variance. *P* < 0.05 was considered statistically significant. 

## 3. Results

### 3.1. Topical Application of Manuka Oil Inhibits UV-B Irradiation-Induced Skin Thickening and Wrinkle Formation in Hairless Mice

**Figure 1:**
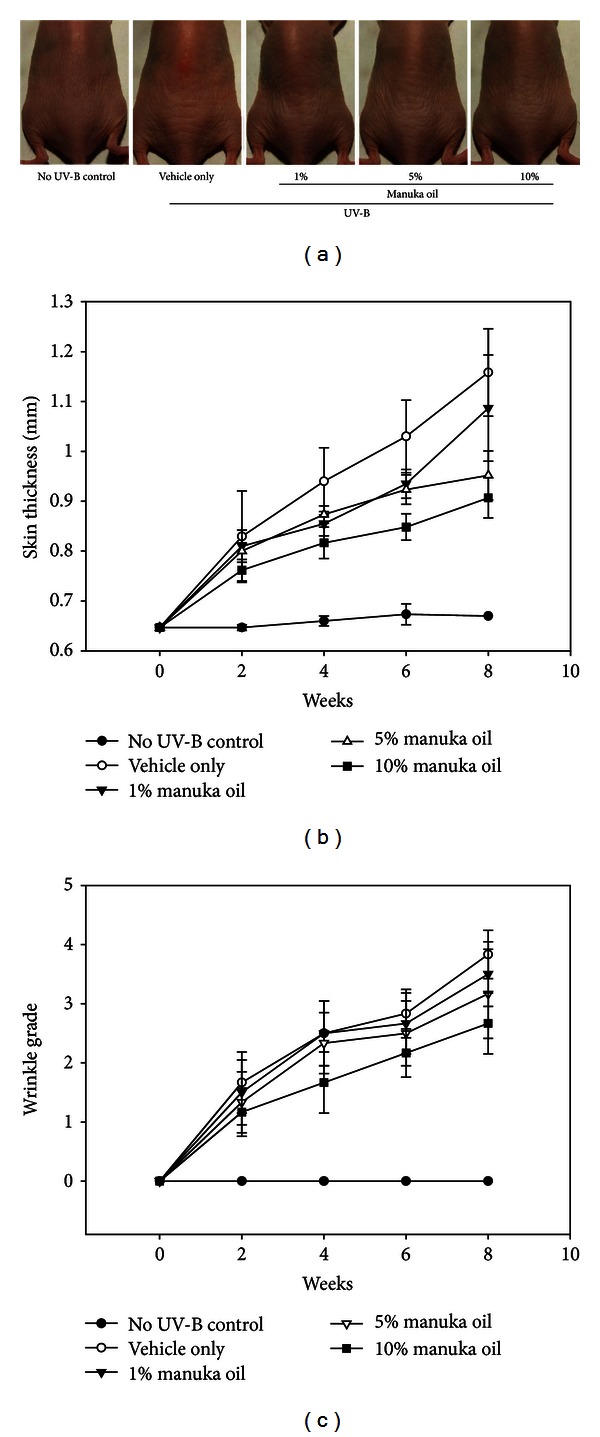
Inhibition of UV-B induced cutaneous photoaging by manuka oil in hairless mice. The dorsal skin surfaces of hairless mice were irradiated by UV-B, 3 times a week for 8 weeks while various concentrations of topical manuka oil were applied every day except Sunday. Each treated group consists of 6 mice. (a) Representative photographs to show the appearance of the dorsal skin area at the end of 8 weeks of the experiment. Increase of skin thickness (b) and wrinkle grade (c) due to repetitive UV-B irradiation and their suppression by manuka oil in a dose-dependent manner. The increases of both skin thickness and wrinkle grade were significantly different between the no UV-B-irradiated control group and the groups treated with 5% and 10% of manuka oil (*P* < 0.01).

In order to evaluate the antiphotoaging activity of manuka oil, we topically applied 150 *μ*L of manuka oil solution diluted with ethanol in 1%, 5%, and 10% concentrations which correspond to doses of 0.09 mg/kg, 0.45 mg/kg, and 0.9 mg/kg, respectively, or a vehicle (ethanol) alone to UV-B-irradiated dorsal skin of hairless mice. In the group treated with the vehicle only, the skin was rough and scaly after 8 weeks of UV-B-irradiation. Furthermore, compared to the nonirradiated control group, the group showed thick and deep wrinkles. Moreover, there were signs of erythema in some mice exposed to UV irradiation. In contrast, following topical application of manuka oil, the visible skin conditions improved in a dose-dependent manner (Figure 1(a)). Repetitive UV irradiation was associated with a gradual increase in skin thickening in the vehicle-treated group, whilst the skin thickness remained almost unchanged in the nonirradiated control group. However, the increase in skin thickness was significantly suppressed in the group treated with manuka oil in a dose-dependent manner (Figure 1(b)). We also estimated the increase in wrinkle formation by using the visual grading method. Repetitive exposure to UV-B radiation increased the wrinkle grade in the mice that were treated with the vehicle alone. However, topical treatment of manuka oil significantly suppressed the increase in the wrinkle grade in a dose-dependent manner (Figure 1(c)). We did not observe any notable phenotypic and behavioral adverse side effects in mice by topical treatment with the manuka oil solutions during the experiment.

**Figure 2:**
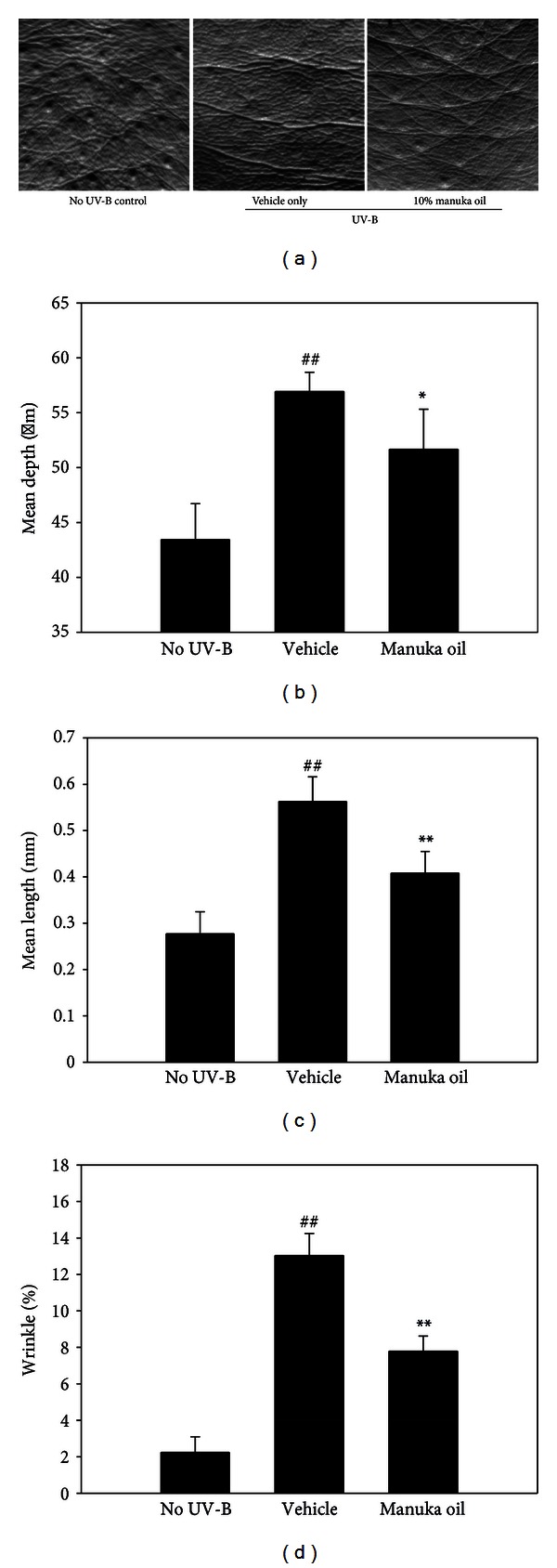
Analysis of wrinkles using skin replicas taken from the dorsal skin. The skin replicas of no UV-B-irradiated control (no UV-B) group, vehicle only-treated group (vehicle), and the group that received 10% manuka oil (manuka oil), respectively, were generated at the end of the experiment. (a) Representative images taken from the replicas. Analysis of the replica images was performed using the Skin-Visiometer and its software for the determination of mean length of wrinkles (b), mean depth of wrinkles (c), and percentage of the wrinkle area (d). Values are means ± SEM (*n* = 6 per group). ^##^: significantly different from no UV-B control group (*P* < 0.01). * and **: significantly different from vehicle only group (**P < 0.01 and *P < 0.05).

The antiwrinkle activity of 10% manuka oil was further analyzed by skin replica method. Repetitive UV-B irradiation for 8 weeks led to the formation of deep and long wrinkles on the skin (Figure 2(a)). However, topical application of 10% manuka oil suppressed the wrinkle formation. When the depth, length, and total % of wrinkles in the images of the replicas were evaluated, significant increases were observed in all 3 the parameters in the vehicle-treated group compared to the nonirradiated control group. However, topical treatment of 10% manuka oil significantly decreased the depth, length, and total % of wrinkles (Figures 2(b), 2(c), and 2(d)).

### 3.2. Manuka Oil Suppresses Epidermal Hyperplasia and Prevents the Loss of Fiber Collagen Content in the Skin of UV-B-Exposed Hairless Mice

**Figure 3:**
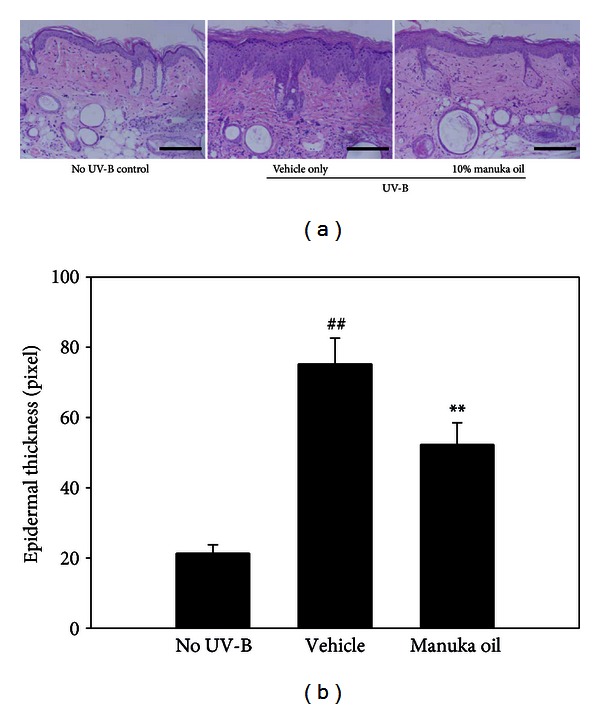
Manuka oil suppresses UV-B-induced increase in epidermal thickness. (a) Representative photographs of haematoxylin and eosin stained dorsal skin sections taken at the end of the experiment from the no UV-B irradiation control (no UV-B) group, vehicle only group (vehicle), and the group that received 10% manuka oil (manuka oil), respectively. Magnification: 200x fold; scale bar: 200 *μ*m. (b) The relative values of epidermal thickness are estimated from the digital photoimages of haematoxylin and eosin stained sections and depicted as mean ± SEM (*n* = 6 per group). ^##^: significantly different from no UV-B control group (*P* < 0.01). **: significantly different from vehicle only group (*P* < 0.01).

The antiphotoaging effect by manuka oil was further evaluated by histological analysis of the biopsied skin specimens after staining with haematoxylin and eosin. The stained sections obtained from the animals treated with the vehicle alone showed a significant increase in epidermal thickness because of UV-B irradiation for 8 weeks (Figures 3(a) and 3(b)). This finding is consistent with the previously reported finding that significant epidermal hyperplasia is induced during photoaging due to UV-B irradiation [8]. However, in the mice treated with 10% topical manuka oil, the epidermal hyperplasia was significantly diminished compared to that in the group treated with the vehicle alone. This result suggests that manuka oil suppresses epidermal hyperplasia to attenuate photoaging.

**Figure 4:**
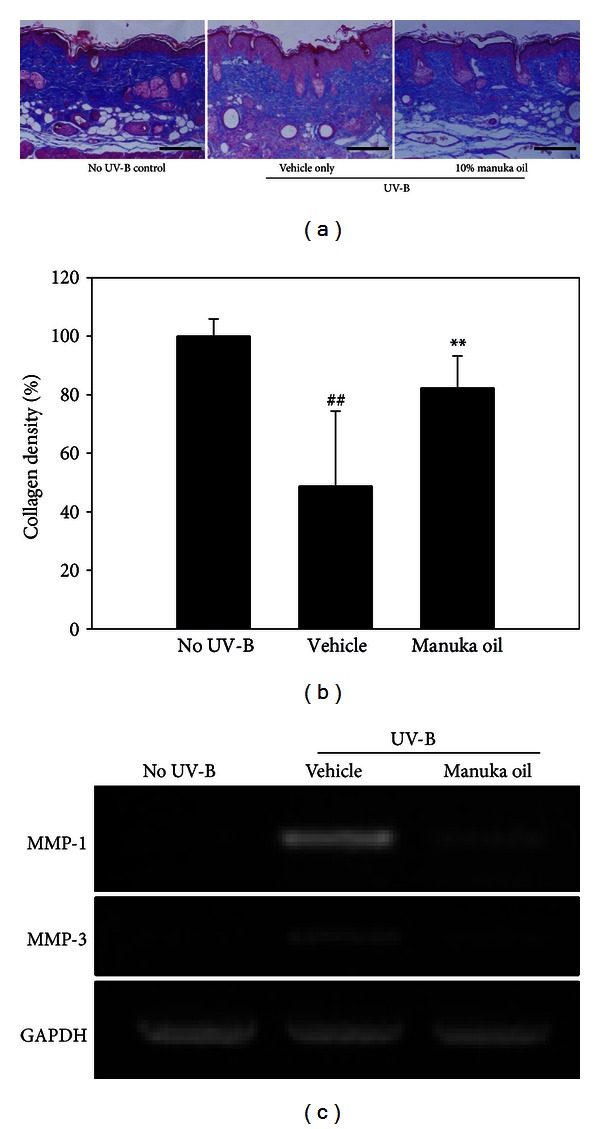
Manuka oil inhibits UV-B-induced reduction of collagen fiber content and expression of MMPs. (a) Representative images of collagen fibers (blue color) stained by Masson-trichrome staining protocol in the skin biopsy obtained at the end of the experiment from the no UV-B irradiation control group (no UV-B), vehicle only group (vehicle), and the group that received 10% manuka oil (manuka oil), respectively. Magnification: 200x fold; scale bar: 200 *μ*m. (b) Relative % collagen density from each image was estimated by using Image Pro Plus 6.0 software.24 and normalizing to the density of normal skin control (100%). The values are mean ± SEM (*n* = 6 per each group). ^##^: significantly different from noUV-B control group (*P* < 0.01). **: significantly different from vehicle only group (*P* < 0.01). (c) m-RNA levels of MMP-1 and MMP-3 in the skin tissue for each group were estimated using RT-PCR of total RNA isolated from the pooled biopsies of each group obtained at the end of the experiment. Et-Br stained bands are shown afterphotopicture. RT-PCR of GAPDH m-RNA was used for an internal control.

We also evaluated the change in collagen content in the skin tissues exposed to UV-B irradiation and the effect of topical treatment of manuka oil on this parameter by using Masson-trichrome staining method. UV-B irradiation for 8 weeks was associated with significantly reduced density of the stained dermal collagen fibers in the tissues treated with the vehicle compared to the normal skin tissues. However, a significantly higher staining density was observed with topical administration of 10% manuka oil compared to vehicle treatment alone (Figures 4(a) and 4(b)). We estimated the expressions of MMP-1 and MMP-3 in UV-B-irradiated skin tissues by using RT-PCR analysis. Induced elevations of MMP-1 and MMP-3 m-RNA were observed by the chronic UV-B irradiation. However, the inductions of both MMP-1 and MMP-3 were notably suppressed when 10% manuka oil was treated topically (Figure 4(c)). This result suggests that manuka oil can suppress UV-B irradiation-induced degradation of collagen fibers and this activity is at least in part associated with its inhibition against the UV-B-induced elevations of metallomatrix proteases to destroy collagen matrix. Therefore, our histological data are consistent with the results from the wrinkle analysis and confirm that manuka oil possesses antiphotoaging activity.

### 3.3. Manuka Oil Inhibits the Production of Proinflammatory Cytokines and Suppresses the Infiltration of Macrophages in UV-B-Irradiated Mouse Skin

**Figure 5:**
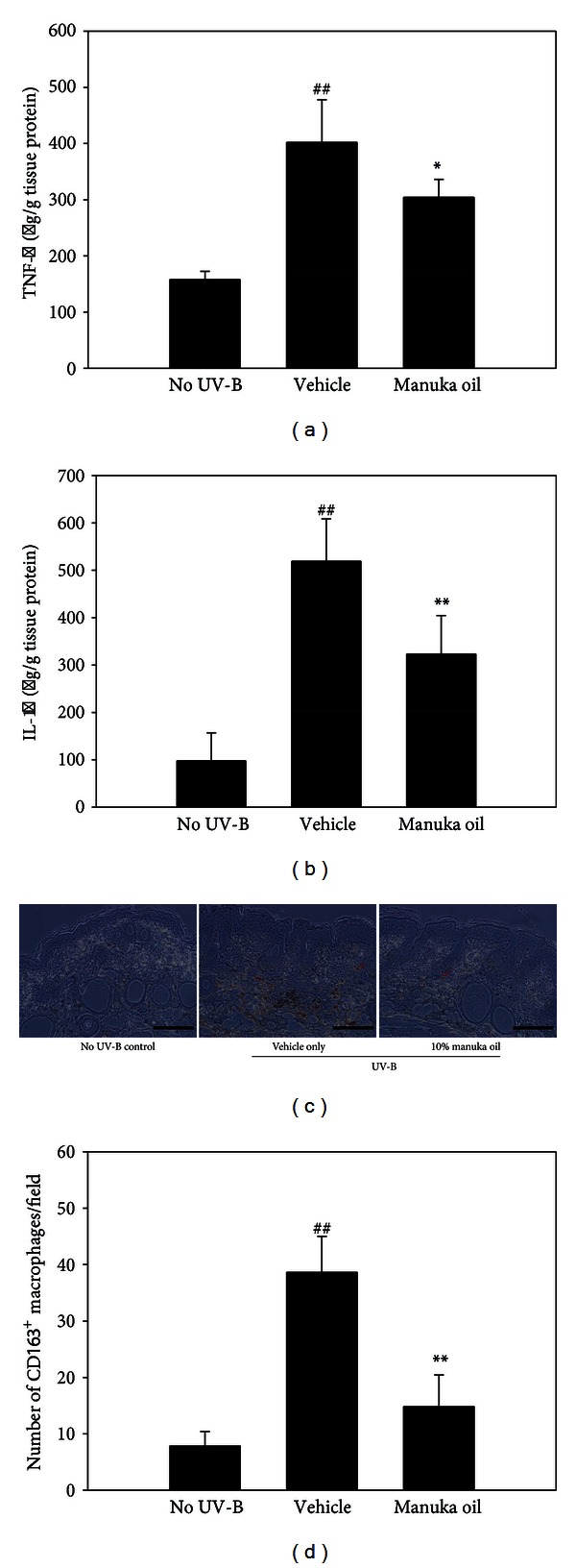
Manuka oil inhibits UV-B-induced production of inflammatory cytokines and infiltration of macrophages. The amount of TNF-*α* (a) and IL-1*β* (b) were estimated using ELISA in the total protein extract of dorsal skin biopsies obtained at the end of the experiment from the no UV-B irradiation control group (no UV-B), vehicle only group (vehicle), and the group that received 10% manuka oil (manuka oil), respectively. Data represent mean ± SEM (*n* = 6 per group). ^##^: significantly different from no UV-B control group (*P* < 0.01). * and **: significantly different from vehicle only group (**P < 0.01 and *P < 0.05). (c) Representative images (×200) from immunohistochemical staining of infiltrated macrophages in dorsal skin biopsies obtained at the end of the experiment. An example of stained macrophage was denoted by a red arrow. Scale bar: 200 *μ*m. (d) Average values ± SEM of macrophage numbers counted per field were represented as a bar graph (*n* = 6 per group).

The production of the proinflammatory cytokines, namely, IL-1*β* and TNF-*α*, was examined in the extract of skin biopsy tissues using ELISA. Eight-week chronic exposure of dorsal skin of hairless mice to UV-B increased the levels of TNF-*α* and IL-1*β* in the tissues treated with the vehicle alone (Figures 5(a) and 5(b)). This result is consistent with the previous report that UV irradiation induces skin inflammation [8, 11]. However, with 10% topical manuka oil, induction of both cytokines was inhibited significantly. In addition, we investigated an extent of macrophage infiltration in the UV-B-irradiated skin by using immunohistochemical staining. The UV-B irradiation for 8 weeks increased the number of macrophages infiltrated into the dermis (Figures 5(c) and 5(d)). However, the topical treatment of 10% manuka oil inhibited the increase significantly by approximately 75.3%. Taken together, these results suggest that topical treatment of manuka oil can suppress UV-B-induced inflammatory reactions in the skin.

## 4. Discussion

Traditionally, various medicinal activities of manuka oil have been well known and studies have provided data to support its bioactivity [2–4, 6]. In recent years, there is a growing interest in the cosmetic properties of manuka oil, as it has been traditionally known to maintain the elasticity of the skin and its youthful appearance. However, there is scarcity of scientific data to support such activities of manuka oil. In this work, we evaluated, for the first time, the protective efficacy of manuka oil against skin photoaging induced by UV-B irradiation with the help of a hairless mouse model. The degree of the activity of different doses of manuka oil was quantified by assessing skin thickness, wrinkle grade, histopathology, and the amount of proinflammatory cytokines. Our work provides clear evidence that the topical application of manuka oil is effective in attenuating skin photoaging. We confirmed that the topical treatment of 10% manuka oil could significantly suppress the signs of photoaging, including wrinkle formation, epidermal thickness, and reduction in collagen fiber content.

We hypothesize the antiphotoaging activity of manuka oil seen in our mouse model study to be due to bioactivities of chemical compounds present in the oil. Firstly, manuka oil contains antioxidant chemicals such as *γ*-terpinene and terpinen-4-ol [2, 3]. These antioxidants are expected to scavenge and directly remove ROS and therefore attenuate the main cause of photoaging induced by chronic UV irradiation. Skin is continuously exposed to environmental insults since it is the first organ to protect the body from environment. UV from sunlight generates reactive oxygen species (ROS) in the skin. ROS in turn induce oxidative stress and skin inflammation giving rise to various symptoms of photoaging. For instance, wrinkle formation, a hallmark of skin aging, occurs because of accumulated skin damages such as matrix destruction and skin inflammation. Therefore, the removal of ROS is a major target to protect skin from solar UV irradiation. Accordingly, the topical application of antioxidants is an approach to ameliorate UV-induced photoaging. In fact, various botanical extracts possessing antioxidant activity were useful in the prevention of UV-B-mediated skin damage [27, 28]. Epigallocatechin gallate (EGCG), an antioxidant included in green tea, prevented degradation of collagen fibers and DNA damage induced by UV radiation [29].

Cellular signaling pathways involved in ROS induced photoaging have been studiedin detail. ROS generated by UV-B activates ERK1/2, p38 MAPK, and JNK signaling in epidermal keratinocyte and dermal fibroblast cells [30–32]. These signaling pathways eventually enhance the transcriptional activities of AP-1 and NF-*κ*B [13, 14, 31]. The 2 activated transcriptional factors enhance the expressions of MMPs, which degrade skin structure by destroying collagen matrix. Therefore, blocking the signaling molecules activated due to the generation of ROS could be an additional strategy to prevent photoaging. Manuka oil contains sesquiterpene hydrocarbons as one of the major components [1–3]. Interestingly, there have been reports that some sesquiterpene compounds can block the signaling pathways involved in oxidative stress [33, 34]. In this work, we showed that the topical treatment of manuka oil inhibited the reduction of fiber collagen density caused by chronic UV-B irradiation, and this was correlated with the suppression of the induced MMP-1 and MMP-3 expressions. This result suggests that the reduction of MMPs might be responsible for the prevention of loss of collagen in the tissue treated with manuka oil. We propose that bioactive chemicals included in manuka oil such as sesquiterpenes could act to block the cell signalings generated by ROS, thus inhibiting the activation of AP-1 and NF-*κ*B which are mainly responsible for the induction of MMPs. It will be interesting to examine if the sesquiterpenes in manuka oil could inhibit the downstream signaling molecules modulated by ROS.

 Our study suggests that manuka oil can suppress skin inflammation reactions in the UV-B-irradiated hairless mouse model. Multiple studies have suggested UV irradiation to induce skin inflammation [8, 35–37]. UV irradiation enhances the synthesis of proinflammatory cytokines in the skin tissue and stimulates the infiltration and activation of inflammatory cells such as neutrophils and macrophages in the skin [36, 37]. UV irradiation of fibroblasts activates AP-1 and NF-*κ*B to induce the production of proinflammatory cytokines such as IL-1*β* and IL-6 [26, 37]. These cytokines then stimulate inflammatory cells such as neutrophils and macrophages in the skin to produce IL-1*β* and TNF-*α*. Eicosanoids such as the prostaglandins and leukotrienes also play a role in provoking skin inflammation and their generation is stimulated under oxidative stress [38].

 Skin inflammation causes premature skin aging because it can increase the expression of MMPs and damage cellular and molecular integrity of the dermis and the epidermis [36]. Accordingly, inhibition of UV-irradiation induced-inflammatory responses is important to protect skin from photoaging. In fact, inhibition of skin inflammation by topical application of anti-inflammatory agents attenuates photoaging [39]. Therefore, we suggest that the inhibitory activity of manuka oil against skin inflammation is crucial for its antiphotoaging efficacy. Further studies would be necessary to identify the chemical components in manuka oil that exert anti-inflammatory activity. We propose antioxidant chemicals in manuka oil to be responsible for the anti-inflammatory activity of the oil. To support this hypothesis, EGCG, a plant antioxidant compound inhibited infiltration of inflammatory cells in UV-irradiated mice skin [40]. It is also possible for the sesquiterpene compounds present in the manuka oil to confer an anti-inflammatory effect. There have been multiple reports suggesting sesquiterpene hydrocarbons to possess anti-inflammatory activity [41–43]. 

A recent report indicated that manuka oil possesses a strong antimicrobial activity against *Propionibacterium acnes*, which suggests that manuka oil could be effective against acne [44]. Along with this, our work suggests that manuka oil is indeed useful in skin care and functional cosmetology.

## 5. Conclusion

We provide evidence that manuka oil can attenuate cutaneous photoaging in a controlled study with the help of UV-B-irradiated hairless mouse model. We suggest antioxidant chemicals and sesquiterpene compounds in manuka oil to underlie such an effect. Therefore, our work supports that manuka oil can be used in the formulation of skin care and functional cosmetic products.
